# Reproducibility of late gadolidium enhancement of atrial ablation scar

**DOI:** 10.1186/1532-429X-18-S1-Q28

**Published:** 2016-01-27

**Authors:** Henry Chubb, Rashid Karim, John Whitaker, James Harrison, Matthew Wright, Mark O'Neill, Reza Razavi

**Affiliations:** 1 Institute of Imaging Sciences and Biomedical Engineering, King's College London, London, United Kingdom; 2 Cardiology, Guy's and St Thomas' NHS Foundation Trust, London, United Kingdom

## Background

Late gadolinium enhancement (LGE) can be used to visualise post-ablation scar in the left atrium, such as following pulmonary isolation procedures for atrial fibrillation. Studies have demonstrated a correlation between ablation site, LGE scar formation and clinical outcomes. However, the reproducibility of scar imaging is undetermined.

## Methods

Eleven patients with atrial fibrillation (AF, 4 persistent) underwent first time pulmonary vein isolation procedure. 3D LGE magnetic resonance imaging (1.5T Philips Ingenia) was performed at 3 months (91 ± 15 days) post ablation (scan 1), with a reproducibility scan three days later (scan 2). 3D LGE acquisitions were performed at 10, 20 and 30 min post gadolinium (Gd, 0.2 mmol/kg) (spatial resolution 1.3 × 1.3 × 4 mm, reconstructed to 0.6 × 0.6 × 2 mm). Scan 2 was performed 48-96 hours after the Scan 1.

The left atrium (LA) was manually segmented and LGE enhancement projected onto the shell (maximum intensity projection technique). Each pulmonary vein (PV) was analysed by 4 independent observers. The percentage of scar encirclement of each PV was quantified visually between 0% (no scar) and 100% (complete encirclement of vein by scar). The LA shell was thresholded at 3.3 standard deviations above the blood pool mean.

## Results

**Figure 1:**
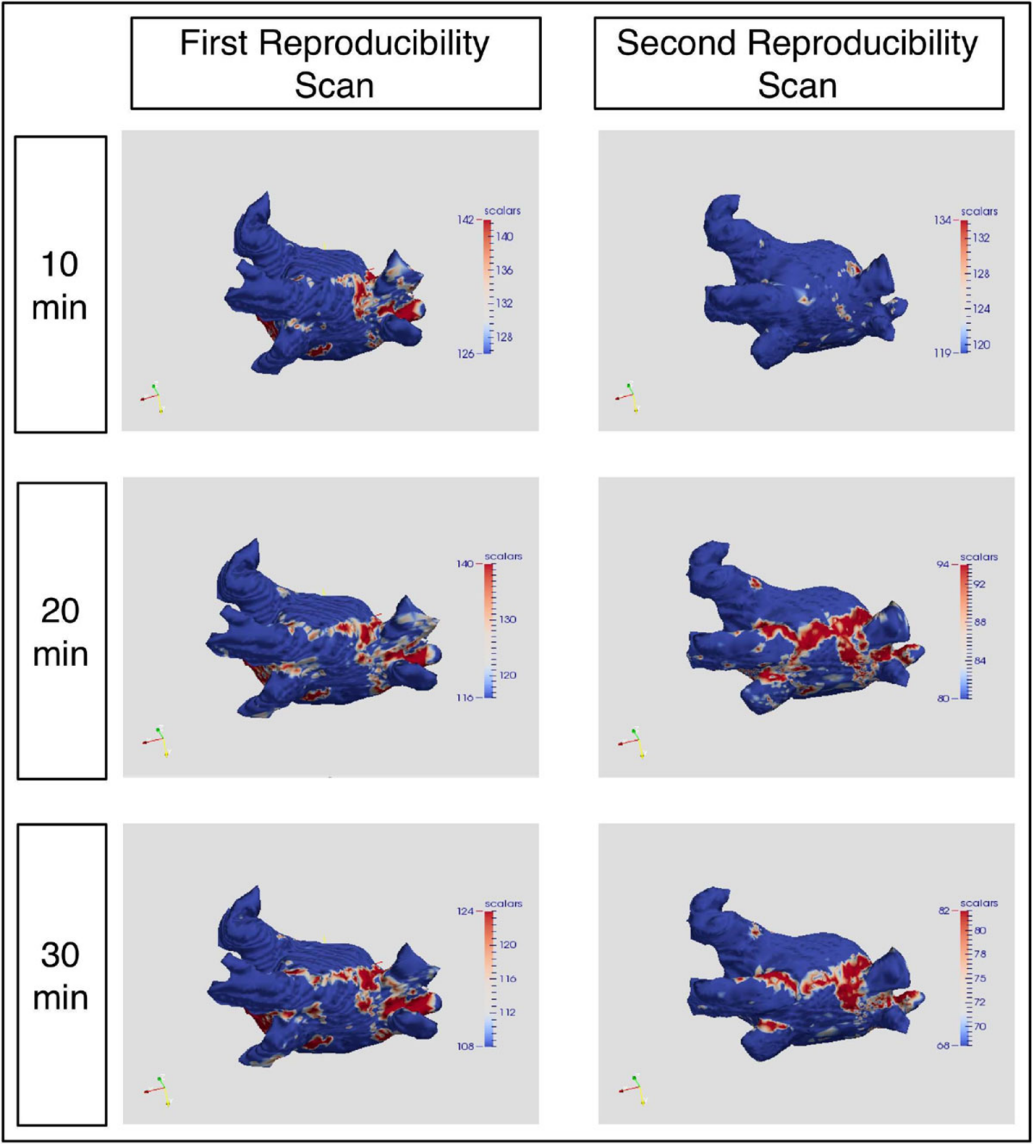
**Left atrial shells generated from 3D LGE acquisition**. Signal intensity derived from maximum intensity projection interogation and shells thresholded at 3.3SD above the blood pool mean.

Sixty six 3D LGE sequences were acquired, totalling 1056 PV measurements (Figure 1). There was good agreement between observers (ICC 0.885 (95% CI 0.85-0.91)). There was significantly less enhancement surrounding the LUPV compared to other veins (p < 0.001, Kruskall-Wallis), and observed PV encirclement was significantly lower at 10 min post Gd than at 20 or 30 min (21 ± 28% vs 69 ± 28%, p < 0.001). Imaging performed 10 min post Gd was therefore not assessed for reproducibility, as it was judged an insensitive measure of scar imaging.

Pulmonary vein encirclement was moderately reproducible. On assessment of maximum encirclement of each individual vein (the higher figure for each vein at either 20 min or 30 min post Gd), mean error between Scan 1 and Scan 2 was 2.0 (± 10.9)%. For mean encirclement, averaging measurements for each vein at 20 min and 30 min, mean error was 0.7% (± 18.4%).

## Conclusions

The utility of LGE assessment of left atrial scar following ablation in guiding further interventions has varied between centres, and a study of the reproducibility of LGE assessment of atrial scar is clearly required. This study suggests that there is a good degree of reproducibility, but that it is highly dependent upon the timing of the acquisition following gadolinium administration. Current data suggests that the main shortfall of LGE scar assessment is one of sensitivity of scar detection, rather than specificity. An approach of multiple acquisitions, and assessment of maximum scar, may be the most reproducible.

